# Supplementary material for: Discovery and Evaluation of Cadmium‐Adapted Daphnia pulex Genotypes in a Region of Historical Mining Reveals Adaptation Protects the Germline From Cadmium‐Induced Mutations
Source: Mol Ecol. 2026 Apr 27;35:e70357. doi: 10.1111/mec.70357 (PMC13113238; doi:10.1111/mec.70357)
Supplement: Supplementary file 1 — Figure S1: Experimental Design (Filename: keith_et_al_cd_adaptation_and_mutatation_supplemental_ReResubmisssion_ME_4‐15‐2026.docx). Figure S2: Hybrid vigour analysis results. Boxplots show the total reproduction of D. pulicaria sires (n = 9), D. pulex dams (n = 4), and their laboratory generated F1 hybrids (n = 15) (clones are from Heier and Dudycha 2009) across exposure to three cadmium concentrations (0, 2.5, and 5 μg/L). Strain was included as a nested random effect in the linear mixed‐effect model and each strain consisted of individual biological replicates (n = 5 per strain). Asterisks (**) denote a significant difference within the 0 μg/L conditions between the F1 hybrids and the mid‐parent mean for the parental strains, indicating hybrid vigour within control conditions (p = 0.006). No significant differences were found between the F1 hybrids and either parental strain or mid‐parent mean in both cadmium concentrations, indicating that general hybrid vigour in control conditions does not confer a specific, enhanced tolerance to cadmium. Figure S3: NONA and ADAP multinucleotide mutations. Figure S4: Regional SNM rates in ADAP experiment. Figure S5: Regional SNM rates in NONA experiment. Figure S6: Total Base Pairs per generation via CNVs (> 3 kb). LIN is a D. pulex clone from Linwood, Ontario. TCO is a D. pulex clone from Slimy Log Pond, Oregon, USA. Figure S7: The number of CNV mutations per generation (CNVs > 3 kb). Table S1: Lake populations and coordinates sampled from Sudbury and Dorset, Ontario, Canada. Sexual or asexual reproduction was determined with the Method outlined by Schaack et al. (2010). Table S2: Microsatellite loci information. Table S3: Clone/Lake, sire and dam information, and lake locations for parental strains and laboratory generated hybrids (denoted as ‘F1’ column one) that were used to test for hybrid vigour in cadmium exposure. Table S4: Genome‐wide mutation rate data for Nonadapted genotype in control conditions. ‘Depth of Coverage’ [file MEC-35-e70357-s001.docx]

**Supplemental Material**

**Figure S1**

**Figure S2**

**
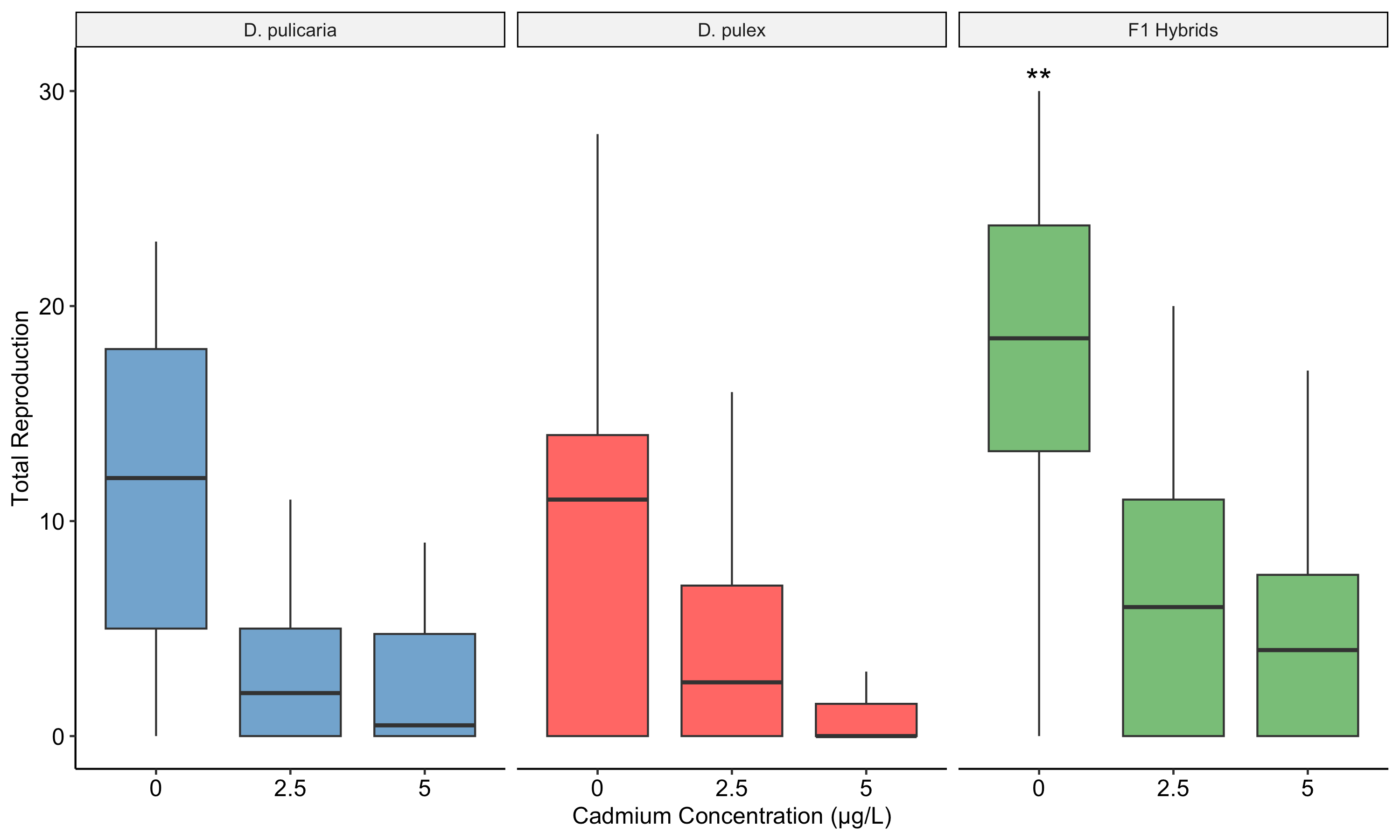
**

Boxplots show the total reproduction of *D. pulicaria* sires (n = 9)*, D. pulex* dams (n = 4)*,* and their laboratory generated F_1_ hybrids (n = 15) (clones are from Heier and Dudycha 2009) across exposure to three cadmium concentrations (0, 2.5, and 5 µg/L). Strain was included as a nested random effect in the linear mixed-effect model and each strain consisted of individual biological replicates (n = 5 per strain). Asterisks (**) denote a significant difference within the 0 µg/L conditions between the F_1_ hybrids and the mid-parent mean for the parental strains, indicating hybrid vigor within control conditions (P = 0.006). No significant differences were found between the F_1_ hybrids and either parental strain or mid-parent mean in both cadmium concentrations, indicating that general hybrid vigor in control conditions does not confer a specific, enhanced tolerance to cadmium.

**Figure S3. NONA and ADAP multinucleotide mutations**


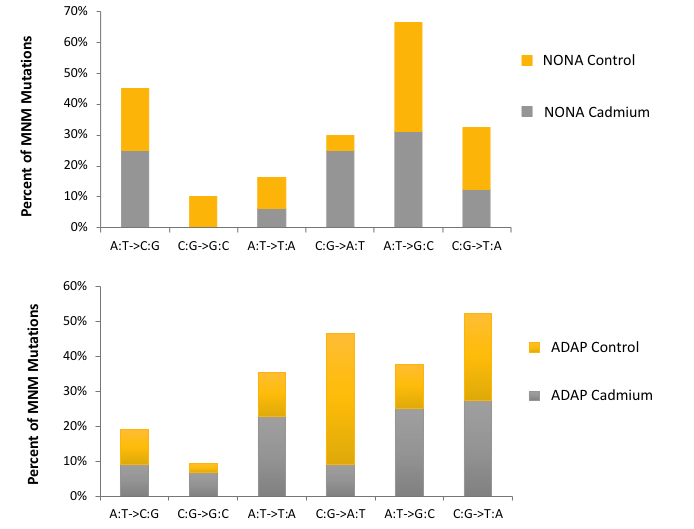


**Figure S4. Regional SNM rates in ADAP experiment**

**Figure S5. Regional SNM rates in NON experiment**

**Figure S6. Total Base Pairs per generation via CNVs (> 3kb). LIN is a *D. pulex* clone from Linwood, Ontario. TCO is a *D. pulex* clone from Slimy Log Pond, Oregon, USA.**

**LIN and TCO (Keith et al. 2016)**

**Figure S7. The number of CNV mutations per generation (CNVs > 3kb)**

**LIN and TCO (Keith et al. 2016)**

**Table S1. Lake populations and coordinates sampled from Sudbury and Dorset, Ontario, Canada. Sexual or asexual reproduction was determined with the Method outlined by Schaack et al. (2010).**

| **Lake** | **Region** | **Latitude** | **Longitude** | **Species** | **Reproduction** |
| --- | --- | --- | --- | --- | --- |
| **Basshaunt** | Dorset | 45° 7'26.38"N | 78°27'47.19"W | *D. pulicaria* | sexual |
| **Brandy** | Dorset | 45° 6'25.74"N | 79°31'35.71"W | *D. pulicaria* | sexual |
| **Buck** | Dorset | 45°23'32.55"N | 78°59'29.87"W | *D. pulicaria* | asexual |
| **Crown** | Dorset | 45°26'6.54"N | 78°40'7.22"W | *D. pulicaria* | sexual |
| **Frenchman** | Sudbury | 46°42'47.78"N | 80°59'6.74"W | *D. pulicaria* | sexual |
| **Glen** | Dorset | 45° 7'53.53"N | 78°28'32.14"W | *D. pulicaria* | sexual |
| **Joe** | Sudbury | 46°44'5.21"N | 81° 0'42.96"W | *D. pulicaria* | sexual |
| **Kelly** | Sudbury | 46°26'48.05"N | 81° 4'0.95"W | *D. pulicaria/D. pulex hybrid* | asexual |
| **Leech** | Dorset | 45° 3'10.12"N | 79° 5'57.60"W | *D. pulicaria* | sexual |
| **MacFarlane** | Sudbury | 46°25'0.79"N | 80°57'43.92"W | *D. pulicaria/D. pulex hybrid* | asexual |
| **McCharles** | Sudbury | 46°22'55.29"N | 81°14'18.36"W | *D. pulicaria/D. pulex hybrid* | asexual |
| **Ramsey** | Sudbury | 46°28'34.15"N | 80°58'38.58"W | *D. pulicaria* | sexual |
| **Simon** | Sudbury | 46°23'53.95"N | 81°11'18.95"W | *D. pulicaria/D. pulex hybrid* | asexual |

**Table S2. Microsatellite loci information**

| **Name** | **Locus** | **NCBI Accession** | **Forward primer** | **Reverse primer** |
| --- | --- | --- | --- | --- |
| Dp140 | P2-G9 | WFms0000144 | AGAGTAACGCGACGGAATGC | CATCGCTTTGCTCTTTTTCCA |
| Dp123 | P2-B17 | WFms0000126 | GGCATCCTCCCAGTAATTGA | TTAGCCAGCCCTCAGAAAAA |
| Dp143 | P2-H21 | WFms0000147 | CTCAGCAACCAGGACCGTTG | ACCTGGAACCTGCAATGACG |
| Dp74 | P1-M20 | WFms0000076 | TGCGCCGCGATGTTTTCC | TGCGACCGACTTATGAACCAACTG |
| Dp28 | P1-C24 | WFms0000028 | GAAGGCGAAACATAAATAAAACAC | AACCCCGGCGTGAATCC |

**Table S3. Clone/Lake, sire and dam information, and lake locations for parental strains and laboratory generated hybrids (denoted as “F1” column one) that were used to test for hybrid vigor in cadmium exposure.**

| Clone/Lake | Species | *pulicaria* Sire | *pulex* Dam | Lake Location |
| --- | --- | --- | --- | --- |
| F1-005 | F1 | Gull 10 | RW 1 | n/a |
| F1-012 | F1 | Warner 5 | West 2 | n/a |
| F1-013 | F1 | Warner 5 | LL3110 | n/a |
| F1-014 | F1 | Lost Creek | Busey 16A | n/a |
| F1-015 | F1 | Lost Creek | Busey 16A | n/a |
| F1-035 | F1 | Warner 5 | LL3110 | n/a |
| F1-038 | F1 | Warner 5 | LL3110 | n/a |
| F1-039 | F1 | Warner 5 | LL3110 | n/a |
| F1-041 | F1 | Warner 2 | Busey 16A | n/a |
| F1-042 | F1 | Warner 2 | Busey 16A | n/a |
| F1-046 | F1 | Fish Lake | POVI 4 | n/a |
| F1-051 | F1 | Fish Lake | West 5 | n/a |
| F1-054 | F1 | Warner 5 | LL3110 | n/a |
| F1-055 | F1 | Warner 5 | LL3110 | n/a |
| F1-056 | F1 | Warner 5 | LL3110 | n/a |
| Fish Lake | *pulicaria* | n/a | n/a | Oregon |
| Gull 10 | *pulicaria* | n/a | n/a | Michigan |
| Little Cultis | *pulicaria* | n/a | n/a | Oregon |
| Lost Creek | *pulicaria* | n/a | n/a | Oregon |
| Pine | *pulicaria* | n/a | n/a | Michigan |
| Warner 14 | *pulicaria* | n/a | n/a | Michigan |
| Warner 17 | *pulicaria* | n/a | n/a | Michigan |
| Warner 2 | *pulicaria* | n/a | n/a | Michigan |
| Warner 5 | *pulicaria* | n/a | n/a | Michigan |
| Busey 16A | *pulex* | n/a | n/a | Illinois |
| POVI 4 | *pulex* | n/a | n/a | Michigan |
| RW2 | *pulex* | n/a | n/a | Michigan |
| West 2 | *pulex* | n/a | n/a | Illinois |

**Table S4. Genome-wide mutation rate data for Nonadapted genotype in control conditions. “**Depth of Coverage” is the genome-wide average depth of sequencing coverage after mapping. Generations is abbreviated as **“**Gens.”. “No. of mutations” is the number of single nucleotide mutations. “Ts/Tv ratio” is the ratio of transitions to transversions. Single nucleotide mutation is abbreviated as “SNM”. “S.E.” is the standard error.

**Table S5. Genome-wide mutation rate data for Adapted genotype in control conditions. “**Depth of Coverage” is the genome-wide average depth of sequencing coverage after mapping. Generations is abbreviated as **“**Gens.”. “No. of mutations” is the number of single nucleotide mutations. “Ts/Tv ratio” is the ratio of transitions to transversions. Single nucleotide mutation is abbreviated as “SNM”. “S.E.” is the standard error.

**Table S6. Conditional mutation rate results for Nonadapted genotype in control conditions**. The overall conditional mutation rate for each mutation class is listed at the bottom of the table, with the S.E. directly below.

**Table S7. Conditional mutation rate results for Adapted genotype in control conditions.**

The overall conditional mutation rate for each mutation class is listed at the bottom of the table, with the S.E. directly below.

**Table S7. Genome-wide mutation rate data for Adapted genotype in cadmium exposure**

**Table S8. Genome-wide mutation rate data for Nonadapted genotype in cadmium exposure. “**Depth of Coverage” is the genome-wide average depth of sequencing coverage after mapping. Generations is abbreviated as **“**Gens.”. “No. of mutations” is the number of single nucleotide mutations. “Ts/Tv ratio” is the ratio of transitions to transversions. Single nucleotide mutation is abbreviated as “SNM”. “S.E.” is the standard error.

**te data for Nonadapted genotype in cadmium exposure. “**Depth of Coverage” is the genome-wide average depth of sequencing coverage after mapping. Generations is abbreviated as **“**Gens.”. “No. of mutations” is the number of single nucleotide mutations. “Ts/Tv ratio” is the ratio of transitions to transversions. Single nucleotide mutation is abbreviated as “SNM”. “S.E.” is the standard error.

**Table S9. Conditional mutation rate results for Nonadapted genotype in cadmium exposure.** The overall conditional mutation rate for each mutation class is listed at the bottom of the table, with the S.E. directly below.

**Table S10. Conditional mutation rate results for Adapted genotype in cadmium exposure.**

The overall conditional mutation rate for each mutation class is listed at the bottom of the table, with the S.E. directly below.

**Table S11. Proportion of total mutations in discrete genome regions compared to the random expectation.**

| **Genotype (Condition)** | **Genome Region** | **Proportion of Mutations** | **P-value** |
| --- | --- | --- | --- |
| NONA (Control) | Intergenic | 0.511 | 0.0727 |
| NONA (Control) | Promoter | 0.042 | 0.4179 |
| **NONA (Control)** | **Exon** | **0.277** | **0.0003***** |
| NONA (Control) | Splice Junction | 0.064 | 0.4686 |
| NONA (Control) | Intron | 0.085 | 0.2791 |
| NONA (Control) | 3-UTR | 0.021 | 0.3114 |
| NONA (Cadmium) | Intergenic | 0.674 | 0.1879 |
| NONA (Cadmium) | Promoter | 0.047 | 0.527 |
| NONA (Cadmium) | Exon | 0.105 | 0.5271 |
| NONA (Cadmium) | Splice Junction | 0.07 | 0.318 |
| NONA (Cadmium) | Intron | 0.105 | 0.6291 |
| NONA (Cadmium) | 3-UTR | 0 | 0.6301 |
| ADAP (Control) | Intergenic | 0.631 | 0.5578 |
| ADAP (Control) | Promoters | 0.081 | 0.6307 |
| ADAP (Control) | Exons | 0.107 | 0.4005 |
| ADAP (Control) | Splice Junctions | 0.081 | 0.0845 |
| ADAP (Control) | Introns | 0.087 | 0.1425 |
| ADAP (Control) | 3' UTRs | 0.013 | 0.7005 |
| ADAP (Cadmium) | Intergenic | 0.558 | 0.2421 |
| ADAP (Cadmium) | Promoters | 0.058 | 0.6549 |
| **ADAP (Cadmium)** | **Exons** | **0.192** | **0.0337*** |
| **ADAP (Cadmium)** | **Splice Junctions** | **0.105** | **0.0022**** |
| ADAP (Cadmium) | Introns | 0.081 | 0.0679 |
| ADAP (Cadmium) | 3' UTRs | 0.006 | 0.728 |

**Table S11. Context-dependent mutation results for Nonadapted and Adapted genotype in control conditions.** “Tot. Trips” is the number of observed triplets analyzed in the genome. “Mut Trips” is the number of observed mutations for each type of triplet. Expected is the expectation if mutations were randomly distributed genome-wide. “CpG” refers to all combined contexts where the site of the mutation was originally a C or G, and was flanked on either or both sides by C or G.

| **Experimental Comparison** | **Genome Region** | **P-value** |
| --- | --- | --- |
| **NONA (Control) vs. NONA (Cd)** | **Intergenic** | **0.0336*** |
|  | Promoters | 1 |
|  | **Exons** | **0.0044**** |
|  | Junctions | 1 |
|  | Introns | 0.7997 |
|  | 3'UTRs | 0.4981 |
| **ADAP (Control) vs. ADAP (Cd)** | Intergenic | 0.2107 |
|  | Promoters | 0.5086 |
|  | **Exons** | **0.0429*** |
|  | Junctions | 0.565 |
|  | Introns | 1 |
|  | 3'UTRs | 0.5987 |
| **NONA (Control) vs. ADAP (Control)** | Intergenic | 0.0821 |
|  | Promoters | 0.2970 |
|  | **Exons** | **0.0009***** |
|  | Junctions | 0.8025 |
|  | Introns | 1 |
|  | 3'UTRs | 0.6416 |
| **NONA (Cd) vs. ADAP (Cd)** | Intergenic | 0.0809 |
|  | Promoters | 0.7796 |
|  | Exons | 0.0769 |
|  | Junctions | 0.4960 |
|  | Introns | 0.6435 |
|  | 3'UTRs | 1 |
| **NONA (Control) vs. ADAP (Cd)** | Intergenic | 0.5201 |
|  | Promoters | 0.7759 |
|  | Exons | 0.124 |
|  | Junctions | 0.371 |
|  | Introns | 1 |
|  | 3'UTRs | 0.2856 |
| **NONA (Cd) vs. ADAP (Control)** | Intergenic | 0.5714 |
|  | Promoters | 0.4241 |
|  | Exons | 1 |
|  | Junctions | 1 |
|  | Introns | 0.6496 |
|  | 3'UTRs | 0.5339 |

**Table S12. Pairwise comparisons of the proportion of total mutations in specific genome regions**

**Table S12. Context-dependent mutation results for Nonadapted and Adapted genotype in control conditions.** “Tot. Trips” is the number of observed triplets analyzed in the genome. “Mut Trips” is the number of observed mutations for each type of triplet. Expected is the expectation if mutations were randomly distributed genome-wide. “CpG” refers to all combined contexts where the site of the mutation was originally a C or G, and was flanked on either or both sides by C or G.

**Table S13. Context-dependent mutation results for Nonadapted and Adapted genotype in cadmium exposure.** “Tot. Trips” is the number of observed triplets analyzed in the genome. “Mut Trips” is the number of observed mutations for each type of triplet. Expected is the expectation if mutations were randomly distributed genome-wide. “CpG” refers to all combined contexts where the site of the mutation was originally a C or G, and was flanked on either or both sides by C or G.

**Table S14. Context-dependent mutation results for Nonadapted and Adapted genotype in cadmium exposure.** “Tot. Trips” is the number of observed triplets analyzed in the genome. “Mut Trips” is the number of observed mutations for each type of triplet. Expected is the expectation if mutations were randomly distributed genome-wide. “CpG” refers to all combined contexts where the site of the mutation was originally a C or G, and was flanked on either or both sides by C or G.

**Table S15. 5-hmC readings for ADAP and NONA at concentrations 0, 0.25. and 20 μg Cd /L**

|  | **ADAP 0** | **ADAP 0.25** | **ADAP 20** | **NONA 0** | **NONA 0.25** | **NONA 20** |
| --- | --- | --- | --- | --- | --- | --- |
| **Rep 1** | 0.259 | 0.249 | 0.260 | debris in well | 0.414 | 0.296 |
| **Rep 2** | 0.258 | 0.165 | 0.151 | 0.679 | 0.140 | 0.424 |
| **Rep 3** | 0.178 | 0.317 | 0.381 | 0.434 | 0.167 | 0.248 |
| **Rep 4** | 0.258 | 0.315 | 0.414 | 0.477 | 0.115 | 0.270 |
| **Avg** | 0.238 | 0.244 | 0.301 | 0.530 | 0.209 | 0.310 |
| **Std Dev** | 0.040 | 0.076 | 0.120 | 0.131 | 0.138 | 0.079 |

**Table S16. Summary CNV information for NONA and ADAP in both controls and cadmium exposure.**

Summary CNV information for NONA Control, NONA Cadmium, ADAP Control, ADAP Cadmium, and CNV findings from Keith et al. (2016) *D. pulex* experiment are listed. From Keith et al., ASEX is an obligate asexual *D. pulex* genotype, and SEX is a cyclical parthenogen (sexual) *D. pulex* genotype.

| **Genotype/Condition** | **No. of CNVs** | **Med. Length** | **Avg. Length** | **Total CNV BPs** | **CNV BPs / gen.** | **CNV events / gen.** |
| --- | --- | --- | --- | --- | --- | --- |
| NONA Control | 10 | 29,750 | 51,700 | 517,000 | 818 | 0.016 |
| NONA Cadmium | 3 | 4,000 | 7,167 | 21,500 | 44 | 0.006 |
| ADAP Control | 70 | 11,500 | 88,029 | 6,161,999 | 8,316 | 0.095 |
| ADAP Cadmium | 59 | 8,500 | 80,212 | 4,732,500 | 4,572 | 0.057 |
| LIN - Keith et al. (2016) | 98 | 17,000 | 90,977 | 8,915,750 | 13,111 | 0.144 |
| TCO - Keith et al. (2016) | 6 | 30,250 | 129,917 | 779,500 | 3,056 | 0.024 |

**Table S17. Genome Coordinates of CNVs**

Sub-line, Chromosome, Scaffold, First Position, Last Position, and CNV Length are listed. Chromosome coordinates are from the TCO genetic map. First Position and Last Position are listed in the 5’ to 3’ orientation on the scaffolds where they are observed. CNVs on chromosomes listed as “n/a” are CNVs on scaffolds that are not mapped to specific chromosomes.

| **Subline** | **Chromosome** | **Scaffold** | **First Position** | **Last Position** | **CNV length** |
| --- | --- | --- | --- | --- | --- |
| NONA Control 2 | 4 | 43 | 803500 | 842500 | 39000 |
| NONA Control 12 | 5 | 131 | 0 | 212500 | 212500 |
| NONA Control 12 | 5 | 131 | 235500 | 274500 | 39000 |
| NONA Control 12 | 5 | 39 | 272000 | 309500 | 37500 |
| NONA Control 12 | 5 | 89 | 5500 | 20000 | 14500 |
| NONA Control 12 | 6 | 47 | 911500 | 916500 | 5000 |
| NONA Control 1 | 8 | 22 | 1177500 | 1181000 | 3500 |
| NONA Control 12 | 8 | 20 | 351000 | 489500 | 138500 |
| NONA Control 3 | 9 | 9 | 620500 | 642500 | 22000 |
| NONA Cd 1 | n/a | 69 | 572000 | 577500 | 5500 |
| NONA Cd 3 | 2 | 1 | 3116500 | 3130000 | 13500 |
| NONA Cd 5 | 11 | 67 | 165500 | 169500 | 4000 |
| NONA Cd 5 | 11 | 67 | 261500 | 265500 | 4000 |
| ADAP Control 9 | 2 | 1 | 32000 | 128000 | 96000 |
| ADAP Control 9 | 2 | 1 | 3243000 | 3751500 | 508500 |
| ADAP Control 9 | 2 | 19 | 86000 | 247500 | 161500 |
| ADAP Control 11 | 2 | 19 | 704500 | 1304000 | 599500 |
| ADAP Control 2 | 3 | 2 | 3093000 | 3096500 | 3500 |
| ADAP Control 10 | 3 | 16 | 518000 | 525500 | 7500 |
| ADAP Control 10 | 3 | 21 | 529500 | 590500 | 61000 |
| ADAP Control 10 | 3 | 21 | 681500 | 688500 | 7000 |
| ADAP Control 10 | 3 | 44 | 6500 | 979500 | 973000 |
| ADAP Control 10 | 3 | 114 | 9000 | 114500 | 105500 |
| ADAP Control 10 | 3 | 114 | 147500 | 347000 | 199500 |
| ADAP Control 10 | 3 | 155 | 54500 | 125000 | 70500 |
| ADAP Control 10 | 3 | 164 | 23000 | 64500 | 41500 |
| ADAP Control 10 | 3 | 164 | 122000 | 127000 | 5000 |
| ADAP Control 10 | 3 | 164 | 147000 | 153000 | 6000 |
| ADAP Control 10 | 3 | 164 | 239500 | 258000 | 18500 |
| ADAP Control 10 | 3 | 174 | 141000 | 145500 | 4500 |
| ADAP Control 10 | 3 | 174 | 166500 | 201500 | 35000 |
| ADAP Control 10 | 3 | 174 | 222000 | 235000 | 13000 |
| ADAP Control 10 | 3 | 178 | 43500 | 58000 | 14500 |
| ADAP Control 10 | 3 | 178 | 85500 | 89500 | 4000 |
| ADAP Control 10 | 3 | 178 | 141500 | 188500 | 47000 |
| ADAP Control 10 | 5 | 15 | 1364000 | 1380500 | 16500 |
| ADAP Control 7 | 5 | 60 | 1 | 617500 | 617499 |
| ADAP Control 1 | 6 | 32 | 82500 | 381500 | 299000 |
| ADAP Control 11 | 6 | 32 | 404000 | 423500 | 19500 |
| ADAP Control 7 | 6 | 32 | 424000 | 428000 | 4000 |
| ADAP Control 10 | 7 | 40 | 42000 | 45000 | 3000 |
| ADAP Control 3 | 7 | 48 | 454500 | 457500 | 3000 |
| ADAP Control 10 | 7 | 82 | 62500 | 69000 | 6500 |
| ADAP Control 10 | 7 | 87 | 386500 | 389500 | 3000 |
| ADAP Control 5 | 8 | 34 | 528500 | 531500 | 3000 |
| ADAP Control 10 | 8 | 57 | 291500 | 296000 | 4500 |
| ADAP Control 6 | 9 | 9 | 1396000 | 1410500 | 14500 |
| ADAP Control 11 | 9 | 41 | 465000 | 470500 | 5500 |
| ADAP Control 10 | 9 | 107 | 4500 | 8000 | 3500 |
| ADAP Control 10 | 10 | 6 | 1873500 | 1888500 | 15000 |
| ADAP Control 10 | 10 | 17 | 0 | 454500 | 454500 |
| ADAP Control 12 | 10 | 26 | 740500 | 853000 | 112500 |
| ADAP Control 12 | 10 | 26 | 1002000 | 1013000 | 11000 |
| ADAP Control 10 | 10 | 72 | 19000 | 384500 | 365500 |
| ADAP Control 5 | 10 | 72 | 396000 | 409000 | 13000 |
| ADAP Control 5 | 10 | 72 | 449500 | 459500 | 10000 |
| ADAP Control 10 | 10 | 72 | 573000 | 642500 | 69500 |
| ADAP Control 10 | 10 | 173 | 43500 | 237500 | 194000 |
| ADAP Control 6 | 11 | 8 | 413000 | 497500 | 84500 |
| ADAP Control 4 | 11 | 10 | 2061000 | 2153500 | 92500 |
| ADAP Control 4 | 11 | 67 | 12500 | 15500 | 3000 |
| ADAP Control 4 | 11 | 67 | 53500 | 57000 | 3500 |
| ADAP Control 4 | 11 | 67 | 77500 | 113000 | 35500 |
| ADAP Control 4 | 11 | 67 | 139000 | 158500 | 19500 |
| ADAP Control 4 | 11 | 67 | 367500 | 713000 | 345500 |
| ADAP Control 9 | 11 | 67 | 367500 | 370500 | 3000 |
| ADAP Control 9 | 11 | 67 | 480500 | 690000 | 209500 |
| ADAP Control 10 | n/a | 50 | 118500 | 122000 | 3500 |
| ADAP Control 10 | n/a | 122 | 255500 | 265000 | 9500 |
| ADAP Control 10 | n/a | 122 | 288500 | 292000 | 3500 |
| ADAP Control 10 | n/a | 122 | 312500 | 323500 | 11000 |
| ADAP Control 10 | n/a | 122 | 371500 | 379000 | 7500 |
| ADAP Control 10 | n/a | 132 | 140000 | 144500 | 4500 |
| ADAP Control 10 | n/a | 132 | 175000 | 183500 | 8500 |
| ADAP Control 10 | n/a | 132 | 211500 | 215000 | 3500 |
| ADAP Control 10 | n/a | 132 | 279000 | 282000 | 3000 |
| ADAP Control 10 | n/a | 147 | 275500 | 287500 | 12000 |
| ADAP Control 10 | n/a | 151 | 125500 | 130000 | 4500 |
| ADAP Control 10 | n/a | 168 | 93000 | 145000 | 52000 |
| ADAP Control 10 | n/a | 168 | 256000 | 261000 | 5000 |
| ADAP Control 10 | n/a | 196 | 32000 | 36000 | 4000 |
| ADAP Cd 6 | 1 | 3 | 3679500 | 3777500 | 98000 |
| ADAP Cd 6 | 1 | 53 | 0 | 504000 | 504000 |
| ADAP Cd 2 | 1 | 66 | 189000 | 221500 | 32500 |
| ADAP Cd 6 | 1 | 66 | 590500 | 807500 | 217000 |
| ADAP Cd 6 | 1 | 106 | 0 | 493500 | 493500 |
| ADAP Cd 2 | 1 | 130 | 116500 | 125000 | 8500 |
| ADAP Cd 6 | 1 | 130 | 139500 | 326000 | 186500 |
| ADAP Cd 4 | 2 | 78 | 299000 | 520500 | 221500 |
| ADAP Cd 4 | 3 | 2 | 966500 | 971000 | 4500 |
| ADAP Cd 2 | 3 | 189 | 112000 | 116000 | 4000 |
| ADAP Cd 11 | 4 | 31 | 497000 | 504000 | 7000 |
| ADAP Cd 11 | 4 | 31 | 543000 | 550500 | 7500 |
| ADAP Cd 11 | 4 | 31 | 577500 | 581000 | 3500 |
| ADAP Cd 11 | 4 | 31 | 594500 | 598500 | 4000 |
| ADAP Cd 11 | 4 | 31 | 1016500 | 1025000 | 8500 |
| ADAP Cd 3 | 5 | 15 | 1378000 | 1382500 | 4500 |
| ADAP Cd 2 | 5 | 37 | 90000 | 93000 | 3000 |
| ADAP Cd 12 | 6 | 32 | 252000 | 446500 | 194500 |
| ADAP Cd 7 | 6 | 32 | 409000 | 412500 | 3500 |
| ADAP Cd 10 | 6 | 92 | 203500 | 206500 | 3000 |
| ADAP Cd 10 | 6 | 92 | 476500 | 501000 | 24500 |
| ADAP Cd 12 | 7 | 4 | 963500 | 1019000 | 55500 |
| ADAP Cd 12 | 7 | 4 | 1140000 | 1162000 | 22000 |
| ADAP Cd 4 | 7 | 18 | 1387500 | 1391000 | 3500 |
| ADAP Cd 8 | 7 | 54 | 626500 | 629500 | 3000 |
| ADAP Cd 7 | 7 | 91 | 110000 | 115500 | 5500 |
| ADAP Cd 10 | 8 | 20 | 215500 | 466000 | 250500 |
| ADAP Cd 4 | 8 | 169 | 109500 | 112500 | 3000 |
| ADAP Cd 4 | 8 | 183 | 41500 | 47500 | 6000 |
| ADAP Cd 1 | 8 | 183 | 58000 | 124000 | 66000 |
| ADAP Cd 1 | 8 | 183 | 143000 | 149500 | 6500 |
| ADAP Cd 11 | 9 | 9 | 264000 | 289000 | 25000 |
| ADAP Cd 7 | 9 | 9 | 477000 | 694500 | 217500 |
| ADAP Cd 6 | 9 | 9 | 1104000 | 1108500 | 4500 |
| ADAP Cd 11 | 9 | 9 | 1140500 | 1269000 | 128500 |
| ADAP Cd 4 | 9 | 9 | 1278500 | 1282000 | 3500 |
| ADAP Cd 11 | 9 | 9 | 1281500 | 1312000 | 30500 |
| ADAP Cd 11 | 9 | 9 | 1329000 | 1374500 | 45500 |
| ADAP Cd 11 | 9 | 14 | 1439500 | 1455500 | 16000 |
| ADAP Cd 7 | 10 | 6 | 2290500 | 2297000 | 6500 |
| ADAP Cd 4 | 10 | 17 | 1518000 | 1522500 | 4500 |
| ADAP Cd 10 | 10 | 36 | 931000 | 934000 | 3000 |
| ADAP Cd 10 | 10 | 49 | 10500 | 16500 | 6000 |
| ADAP Cd 6 | 10 | 56 | 114000 | 496500 | 382500 |
| ADAP Cd 2 | 10 | 72 | 390000 | 573000 | 183000 |
| ADAP Cd 4 | 10 | 72 | 452500 | 455500 | 3000 |
| ADAP Cd 4 | 10 | 72 | 573000 | 590500 | 17500 |
| ADAP Cd 2 | 10 | 72 | 625500 | 628500 | 3000 |
| ADAP Cd 6 | 10 | 103 | 57500 | 60500 | 3000 |
| ADAP Cd 6 | 10 | 103 | 271500 | 275000 | 3500 |
| ADAP Cd 1 | 11 | 10 | 1345000 | 2146000 | 801000 |
| ADAP Cd 12 | 11 | 67 | 367000 | 370000 | 3000 |
| ADAP Cd 5 | 11 | 67 | 436000 | 470500 | 34500 |
| ADAP Cd 12 | 11 | 67 | 489500 | 713000 | 223500 |
| ADAP Cd 3 | 12 | 58 | 710000 | 724500 | 14500 |
| ADAP Cd 10 | 12 | 70 | 109500 | 194000 | 84500 |
| ADAP Cd 10 | 12 | 96 | 335500 | 351500 | 16000 |
| ADAP Cd 1 | n/a | 85 | 295000 | 298000 | 3000 |
| ADAP Cd 10 | n/a | 167 | 47500 | 58500 | 11000 |

**Table S18. qPCR Results for genomic DNA (gDNA Gene Amplicon Copies) and RNA (RNA Expression qPCR)**

| **Group** | **Genotype ID** | **Ct (dRN)** | **Copies (ng)** |
| --- | --- | --- | --- |
| Adapted | K10 | 20.62 | 18,070.00 |
| Adapted | K10 | 20.88 | 15,070.00 |
| Adapted | K10 | 20.94 | 14,450.00 |
| Adapted | K10 | 20.95 | 14,370.00 |
| Adapted | K10 | 21.11 | 12,860.00 |
| Adapted | K10 | 21.12 | 12,750.00 |
| Adapted | K2 | 18.51 | 76,930.00 |
| Adapted | K2 | 18.51 | 76,690.00 |
| Adapted | K2 | 18.57 | 73,640.00 |
| Adapted | K2 | 18.59 | 72,700.00 |
| Adapted | K2 | 18.65 | 69,640.00 |
| Adapted | K2 | 18.66 | 69,230.00 |
| Adapted | K3 | 21.03 | 13,610.00 |
| Adapted | K3 | 21.10 | 12,930.00 |
| Adapted | K3 | 21.16 | 12,420.00 |
| Adapted | K3 | 21.33 | 11,050.00 |
| Adapted | K3 | 21.35 | 10,890.00 |
| Adapted | K3 | 21.44 | 10,300.00 |
| Adapted | K9 | 18.60 | 72,410.00 |
| Adapted | K9 | 18.62 | 71,360.00 |
| Adapted | K9 | 18.68 | 68,500.00 |
| Adapted | K9 | 18.85 | 60,800.00 |
| Adapted | K9 | 18.86 | 60,300.00 |
| Adapted | K9 | 18.97 | 56,070.00 |
| Adapted | MC13 | 18.60 | 72,290.00 |
| Adapted | MC13 | 18.62 | 71,360.00 |
| Adapted | MC13 | 18.63 | 70,810.00 |
| Adapted | MC13 | 18.63 | 70,840.00 |
| Adapted | MC14 | 18.15 | 85,300.00 |
| Adapted | MC14 | 18.21 | 81,460.00 |
| Adapted | MC14 | 18.22 | 80,950.00 |
| Adapted | MC14 | 18.26 | 78,680.00 |
| Adapted | MC14 | 18.33 | 75,400.00 |
| Adapted | MC14 | 18.47 | 68,080.00 |
| Adapted | MC8 | 18.57 | 73,750.00 |
| Adapted | MC8 | 18.58 | 72,980.00 |
| Adapted | MC8 | 18.63 | 70,580.00 |
| Adapted | MC8 | 18.63 | 70,840.00 |
| Adapted | MC8 | 18.66 | 69,280.00 |
| Adapted | MF4 | 18.13 | 86,450.00 |
| Adapted | MF4 | 18.17 | 83,800.00 |
| Adapted | MF4 | 18.30 | 76,490.00 |
| Adapted | MF4 | 18.31 | 75,990.00 |
| Adapted | MF4 | 18.41 | 71,220.00 |
| Adapted | MF4 | 18.55 | 64,380.00 |
| Adapted | MF6 | 18.48 | 67,860.00 |
| Adapted | MF6 | 18.49 | 67,270.00 |
| Adapted | MF6 | 18.50 | 66,690.00 |
| Adapted | MF6 | 18.51 | 66,370.00 |
| Adapted | MF6 | 18.52 | 66,110.00 |
| Adapted | S1 | 18.06 | 90,260.00 |
| Adapted | S1 | 18.09 | 88,750.00 |
| Adapted | S1 | 18.14 | 85,710.00 |
| Adapted | S1 | 18.15 | 85,350.00 |
| Adapted | S1 | 18.17 | 83,770.00 |
| Adapted | S14 | 17.94 | 98,330.00 |
| Adapted | S14 | 17.96 | 97,290.00 |
| Adapted | S14 | 18.11 | 87,210.00 |
| Adapted | S9 | 19.49 | 33,800.00 |
| Adapted | S9 | 19.51 | 33,370.00 |
| Adapted | S9 | 19.59 | 31,500.00 |
| Adapted | S9 | 19.61 | 31,070.00 |
| Adapted | S9 | 19.66 | 30,020.00 |
| Adapted | S9 | 19.66 | 29,970.00 |
| Non-Adapted | BH14 | 21.97 | 7,120.00 |
| Non-Adapted | BH14 | 22.03 | 6,841.00 |
| Non-Adapted | BH14 | 22.04 | 6,814.00 |
| Non-Adapted | BH14 | 22.06 | 6,695.00 |
| Non-Adapted | BH14 | 22.25 | 5,886.00 |
| Non-Adapted | BH14 | 22.52 | 4,889.00 |
| Non-Adapted | BH15 | 18.81 | 62,480.00 |
| Non-Adapted | BH15 | 18.83 | 61,520.00 |
| Non-Adapted | BH15 | 18.92 | 58,050.00 |
| Non-Adapted | BH15 | 19.01 | 54,590.00 |
| Non-Adapted | BH15 | 19.06 | 52,460.00 |
| Non-Adapted | BH3 | 18.83 | 61,780.00 |
| Non-Adapted | BH3 | 18.89 | 59,260.00 |
| Non-Adapted | BH3 | 18.91 | 58,540.00 |
| Non-Adapted | BH3 | 18.97 | 56,140.00 |
| Non-Adapted | BH3 | 18.98 | 55,770.00 |
| Non-Adapted | BH3 | 19.00 | 55,030.00 |
| Non-Adapted | BR1 | 19.60 | 36,280.00 |
| Non-Adapted | BR1 | 19.70 | 33,930.00 |
| Non-Adapted | BR1 | 19.70 | 33,950.00 |
| Non-Adapted | BR1 | 19.82 | 31,310.00 |
| Non-Adapted | BR1 | 19.85 | 30,690.00 |
| Non-Adapted | BR16 | 19.59 | 36,500.00 |
| Non-Adapted | BR16 | 19.60 | 36,280.00 |
| Non-Adapted | BR16 | 19.65 | 35,200.00 |
| Non-Adapted | BR16 | 19.66 | 34,760.00 |
| Non-Adapted | BR16 | 19.68 | 34,440.00 |
| Non-Adapted | BR16 | 19.80 | 31,770.00 |
| Non-Adapted | BU12 | 19.16 | 49,300.00 |
| Non-Adapted | BU12 | 19.22 | 47,180.00 |
| Non-Adapted | BU12 | 19.22 | 47,200.00 |
| Non-Adapted | BU12 | 19.25 | 46,250.00 |
| Non-Adapted | BU12 | 19.32 | 44,090.00 |
| Non-Adapted | BU12 | 19.45 | 40,290.00 |
| Non-Adapted | F10 | 18.16 | 84,290.00 |
| Non-Adapted | F10 | 18.22 | 81,140.00 |
| Non-Adapted | F10 | 18.25 | 79,290.00 |
| Non-Adapted | F10 | 18.26 | 78,880.00 |
| Non-Adapted | F10 | 18.29 | 77,420.00 |
| Non-Adapted | F10 | 18.29 | 77,160.00 |
| Non-Adapted | J3 | 18.11 | 87,720.00 |
| Non-Adapted | J3 | 18.11 | 87,540.00 |
| Non-Adapted | J3 | 18.13 | 86,140.00 |
| Non-Adapted | J3 | 18.36 | 73,460.00 |
| Non-Adapted | J3 | 18.41 | 71,090.00 |
| Non-Adapted | J4 | 18.45 | 68,970.00 |
| Non-Adapted | J4 | 18.51 | 66,310.00 |
| Non-Adapted | J4 | 18.65 | 60,290.00 |
| Non-Adapted | J4 | 18.82 | 53,520.00 |
| Non-Adapted | J4 | 18.85 | 52,680.00 |
| Non-Adapted | G11 | 22.10 | 5,576.00 |
| Non-Adapted | G11 | 22.22 | 5,132.00 |
| Non-Adapted | G11 | 22.34 | 4,737.00 |
| Non-Adapted | G11 | 22.54 | 4,105.00 |
| Non-Adapted | G11 | 23.00 | 3,006.00 |
| Non-Adapted | G11 | 23.39 | 2,297.00 |
| Non-Adapted | L7 | 18.29 | 77,470.00 |
| Non-Adapted | L7 | 18.34 | 74,910.00 |
| Non-Adapted | L7 | 18.36 | 73,430.00 |
| Non-Adapted | L7 | 18.45 | 69,010.00 |
| Non-Adapted | L7 | 18.45 | 69,080.00 |
| Non-Adapted | L7 | 18.48 | 67,680.00 |
| Non-Adapted | R15 | 22.87 | 3,288.00 |
| Non-Adapted | R15 | 22.91 | 3,183.00 |
| Non-Adapted | R15 | 22.93 | 3,144.00 |
| Non-Adapted | R15 | 23.01 | 2,987.00 |
| Non-Adapted | R15 | 23.09 | 2,810.00 |
| Non-Adapted | TCO | 18.37 | 73,240.00 |
| Non-Adapted | TCO | 18.42 | 70,830.00 |
| Non-Adapted | TCO | 18.46 | 68,830.00 |

**Table S19. RT-qPCR Results for RNA expression – Adapted vs. Nonadapted**

| **Genotype** | Class | **0 Cd** | **20 Cd** |
| --- | --- | --- | --- |
| K3 | Adapted | 2.9 | 20.67 |
| K3 | Adapted | 2.87 | 22.66 |
| K3 | Adapted | 3.6 | 22.8 |
| K7 | Adapted | 7.03 | 8.35 |
| K7 | Adapted | 6.97 | 7.98 |
| K7 | Adapted | 7.54 | 8.09 |
| K13 | Adapted | 7.41 | 7 |
| K13 | Adapted | 7.61 | 6.99 |
| K13 | Adapted | 6.15 | 7.87 |
| MC8 | Adapted | 8 | 8.93 |
| MC8 | Adapted | 8.35 | 10.08 |
| MC8 | Adapted | 8.62 | 8.63 |
| MF6 | Adapted | 7.58 | 16.44 |
| MF6 | Adapted | 8.41 | 15.79 |
| MF6 | Adapted | 9.19 | 15.59 |
| S9 | Adapted | 5.44 | 13.41 |
| S9 | Adapted | 5.9 | 13.19 |
| S9 | Adapted | 5.45 | 11.15 |
| S14 | Adapted | 9.11 | 18.19 |
| S14 | Adapted | 9.74 | 19.28 |
| S14 | Adapted | 10.06 | 20.34 |
| MC14 | Adapted | 9.71 | - |
| MC14 | Adapted | 7.24 | - |
| MC14 | Adapted | 8.9 | - |
| MF4 | Adapted | 6.01 | - |
| MF4 | Adapted | 5.97 | - |
| MF4 | Adapted | 4.47 | - |
| K3 | Adapted | 3.52 | 25.02 |
| K3 | Adapted | 4.5 | 22.96 |
| K3 | Adapted | 4.41 | 25.95 |
| BH3 | Nonadapted | 5.21 | 29.15 |
| BH3 | Nonadapted | 4.78 | 26.58 |
| BH3 | Nonadapted | 5.11 | 26.61 |
| BH15 | Nonadapted | 4.34 | 13.07 |
| BH15 | Nonadapted | 4.6 | 13.03 |
| BH15 | Nonadapted | 4.36 | 11.74 |
| BR1 | Nonadapted | 6.58 | 10.08 |
| BR1 | Nonadapted | 7.58 | 10.96 |
| BR1 | Nonadapted | 7.84 | 11.29 |
| BR16 | Nonadapted | 7.95 | 13.86 |
| BR16 | Nonadapted | 7.9 | 15.84 |
| BR16 | Nonadapted | 6.82 | 12.98 |
| J3 | Nonadapted | 3.21 | 14.81 |
| J3 | Nonadapted | 3.21 | 14.46 |
| J3 | Nonadapted | 3.39 | 13.83 |
| BU4 | Nonadapted | 1.41 | 9.66 |
| BU4 | Nonadapted | 1.44 | 11.11 |
| BU4 | Nonadapted | 1.58 | 11.54 |
| F10 | Nonadapted | 3.12 | 37.03 |
| F10 | Nonadapted | 2.82 | 39.92 |
| F10 | Nonadapted | 2.57 | 35.47 |
| BH14 | Nonadapted | - | 30.19 |
| BH14 | Nonadapted | - | 30.62 |
| BH14 | Nonadapted | - | 26.64 |
| BR1 | Nonadapted | 5.14 | - |
| BR1 | Nonadapted | 3.53 | - |
| BR1 | Nonadapted | 6.16 | - |
| G11 | Nonadapted | 1.6 | 11.22 |
| G11 | Nonadapted | 1.47 | 10.93 |
| G11 |  | 1.38 | 11.5 |
